# The new role of diagnostic angiography in coronary physiological assessment

**DOI:** 10.1136/heartjnl-2020-318289

**Published:** 2021-01-08

**Authors:** Mina Ghobrial, Hazel Arfah Haley, Rebecca Gosling, Vignesh Rammohan, Patricia V Lawford, D Rod Hose, Julian P Gunn, Paul D Morris

**Affiliations:** 1 Mathematical Modellling in Medicine, Department of Infection Immunity and Cardiovascular Disease, The Medical School, University of Sheffield, Sheffield, UK; 2 Department of Cardiology, Sheffield Teaching Hospitals, Sheffield, UK; 3 Insigneo, In Silico Medicine, University of Sheffield, Sheffield, UK

**Keywords:** cardiac catheterisation, coronary angiography, computed tomography angiography, coronary artery disease

## Abstract

The role of ‘stand-alone’ coronary angiography (CAG) in the management of patients with chronic coronary syndromes is the subject of debate, with arguments for its replacement with CT angiography on the one hand and its confinement to the interventional cardiac catheter laboratory on the other. Nevertheless, it remains the standard of care in most centres. Recently, computational methods have been developed in which the laws of fluid dynamics can be applied to angiographic images to yield ‘virtual’ (computed) measures of blood flow, such as fractional flow reserve. Together with the CAG itself, this technology can provide an ‘all-in-one’ anatomical and functional investigation, which is particularly useful in the case of borderline lesions. It can add to the diagnostic value of CAG by providing increased precision and reduce the need for further non-invasive and functional tests of ischaemia, at minimal cost. In this paper, we place this technology in context, with emphasis on its potential to become established in the diagnostic workup of patients with suspected coronary artery disease, particularly in the non-interventional setting. We discuss the derivation and reliability of angiographically derived fractional flow reserve (CAG-FFR) as well as its limitations and how CAG-FFR could be integrated within existing national guidance. The assessment of coronary physiology may no longer be the preserve of the interventional cardiologist.

## Introduction: Coronary angiography (CAG) in the 2020s

**Figure 1:**
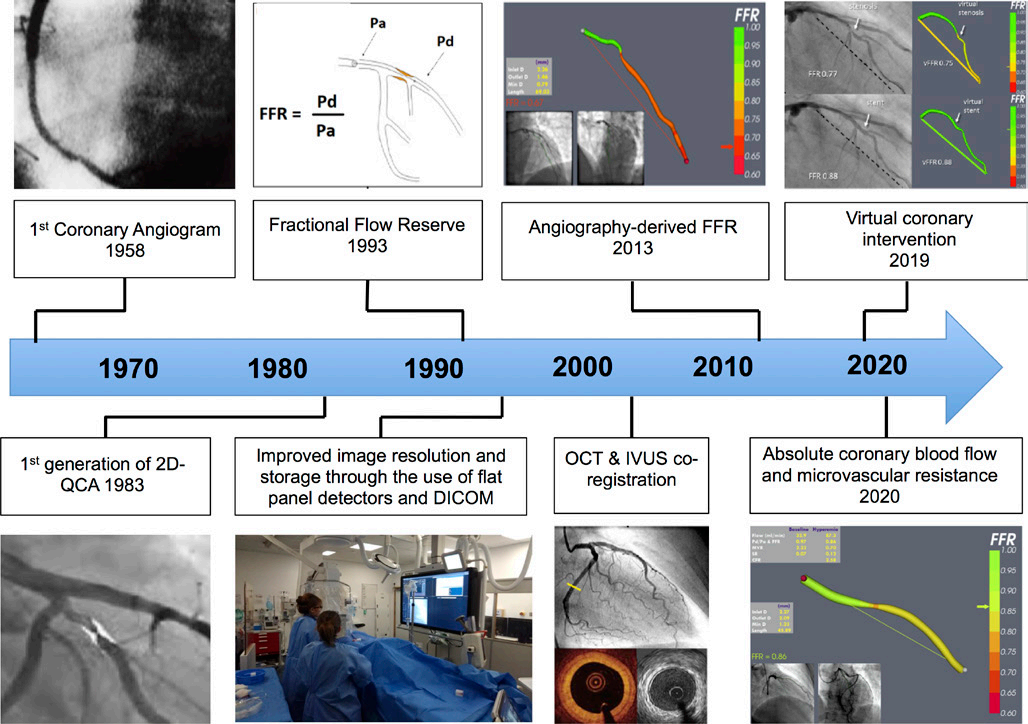
Milestones in the history of diagnostic coronary angiography. 2D, two-dimensional; FFR, fractional flow reserve; IVUS, intravascular ultrasound; OCT, optical coherence tomography; Pa, aortic pressure; Pd, pressure distal to stenosis; QCA, quantitative coronary angiography. DICOM, Digital Imaging and Communications in Medicine

Chronic coronary artery disease (CAD) remains a significant healthcare burden fuelled by greater longevity, increased expectations and high-quality management of acute coronary syndromes. In the UK, the National Institute for Health and Care Excellence guidelines recommend that CT coronary angiography (CTCA) should be offered as the first-line investigation for the investigation of stable chest pain.^1^ According to current activity, this would require an eightfold increase in national service provision.^2^ Approximately 250 000 CAGs, including about 40 000 in non-interventional cardiac catheter laboratories (CCLs), are carried out in the UK per annum, a consistent figure in recent years.^3^ The impact of CTCA on this figure is as yet unclear. Some have suggested that the rise in popularity, accuracy and accessibility of CTCA may signal the death knell for CAG,^4^ yet data reveal a slow increase in numbers.^3^ The main problem with CAG is its invasive nature, although radial artery access, small diameter catheters and improved X-ray contrast medium have reduced the complication rate to negligible levels. Its main diagnostic deficiencies include its anatomical rather than functional nature, a poor relationship between per cent stenosis and blood flow, the subjectivity of visual interpretation particularly in intermediate (30%–70%) stenoses^5^ and technical inadequacies, such as poor vessel opacification and lesion assessment. Nevertheless, CAG remains the final common pathway for revascularisation and treatment planning and is a prerequisite for valve surgery and other major interventions, such as organ transplantation. It is often performed in CCLs, which lack the capability to assess coronary blood flow. Figure 1 illustrates the major milestones in its evolution.

## Fractional flow reserve (FFR)

Coronary blood flow has to adapt to the demands of exercise, which is achieved by reduction in the coronary microvascular resistance. Maximum flow (hyperaemia) is limited by the presence of an epicardial coronary stenosis and impaired microvascular function.^6^ Direct measurement of coronary blood flow is difficult, and the best and most widely used surrogate is FFR, the ratio of distal to proximal translesional pressure, measured with a pressure-sensitive wire during maximum hyperaemia, which is usually induced with an infusion of adenosine. It represents the maximally achievable flow in a stenotic artery as a percentage of the maximum flow expected in the hypothetical absence of that stenosis.^6^ A significant impairment of hyperaemic flow is defined as ≥20% reduction, that is, FFR≤0.80. FFR was originally validated against standard non-invasive tests of ischaemia and the threshold for treatment eventually settled at ≤0.80.^7^

### What does FFR mean?

There is a considerable evidence base and a class 1a recommendation for using FFR, or related indices, to guide percutaneous revascularisation.^8^ This is because most of the evidence for the benefit of FFR is derived from the *interventional* rather than the *initial* stage of management, partly because a pressure wire is, in fact, a modified angioplasty guidewire.^6^ Using FFR to limit percutaneous coronary intervention (PCI) to lesions with FFR of ≤0.80 reduces morbidity and inappropriate revascularisation, with attendant economic benefit, compared with angiographic guidance alone.^9–11^ However, FFR is a continuous variable, so the 0.80 threshold is only the optimal trade-off between sensitivity and specificity at a population level.^12^ At the extremes of FFR (severely stenosed vs nearly normal), there is >95% certainty of clinical decision-making, corresponding with the greatest prognostic and symptomatic benefits, but diagnostic certainty falls to 50% at FFR 0.80.^13^ FFR is, of course, most useful in the mid-zone in cases with moderate stenosis.^14^ In the landmark trials, however, the mean FFR was 0.56–0.68, which is considerably lower than this.^9 10^ Also, there are many factors to be considered when intervening on a lesion, such as its complexity, the size and quality of the distal vessel, the extent of myocardium at risk, other disease and the likely benefit, so decision-making tends to incorporate the FFR (or computed FFR) rather than rely on it entirely.^12^

### How does FFR affect contemporary clinical decision-making?

The RIPCORD (Does routine pressure wire assessment influence management strategy at coronary angiography for diagnosis of chest pain?) study was a UK-based, multicentre, prospective, randomised controlled trial investigating whether incorporating routine FFR measurement at diagnostic angiography in the assessment of stable CAD would result in a change in management compared with angiographic assessment alone. The primary endpoint was the difference in management plan per coronary artery between the one made using the angiogram alone versus after disclosure of the FFR. Two hundred and three patients were randomised. There was a change in management plan after FFR was disclosed in 53 (26%) patients, and the number and location of significant stenoses changed in 64 (32%). Of 72 cases in which optimal medical therapy (OMT) was initially recommended after CAG, nine (13%) were physiologically significant at FFR and were therefore referred for revascularisation. Conversely, of 89 cases in whom the management plan was OMT based on FFR, revascularisation would have been recommended in 25 (28%) based on CAG only.^15^ Similar influences of FFR on angiographic decision-making were also observed in the setting of acute coronary syndromes in the FAMOUS-NSTEMI (Fractional flow reserve vs angiography in guiding management to optimize outcomes in non-ST-segment elevation myocardial infarction) study, which reported a change in management strategy in 21.6% of patients, resulting in fewer procedures and unplanned revascularisations.^16^ These studies underline the importance of physiological guidance in everyday contemporary practice at the time of CAG.

## Coronary artery bypass surgery (CABG): A major unmet need for physiological guidance

The patients most deserving of physiological guidance are those with multivessel disease being considered for CABG.^17^ However, very few receive pressure wire assessment prior to CABG, and often, referral for surgery is based on a CAG performed in a non-interventional CCL. Further guidance with FFR would require a second visit to a CCL, with associated delays, and is therefore rarely done. Without FFR, if two vessels need grafting, the third being angiographically borderline, the surgeon may feel obliged to apply a graft which, if the lesion is physiologically insignificant, may lead to an unnecessarily long operation, a wasted conduit, and occlusion due to competitive flow. Anatomical triple vessel disease, ‘mandating’ CABG, when subjected to physiological assessment, may be converted to physiological two- or even one-vessel disease, adequately treated by PCI. This was described in a subanalysis of the SYNTAX (Taxus drug-eluting stent versus coronary artery bypass surgey for the treatment of narrowed arteries) II trial in which only 37.2% of patients remained being classified as having triple vessel disease after invasive physiological assessment.^18^ This group of patients may derive particular benefit from angiography-derived FFR (FFR computed from the angiogram). Although clinical trials comparing physiology-guided CABG with angiography-guided CABG have not shown clear benefit of physiological guidance, these trials included small numbers of patients, and it seems counterintuitive to graft a vessel with non-flow-limiting disease.^19^ Retrospective analysis of the major revascualrisation trials using CAG-FFR is limited due to the lack of a specific acquisition protocol required to fulfil all the technical requirements of CAG-FFR analysis.^20^

## Why is FFR so rarely employed?

In view of the universally beneficial influence of FFR in CAD, it is surprising that its use, even in the setting of PCI, is so sparse.^5^ The reasons are relevant to the future uptake of angiography-derived FFR. First, measured physiology, requiring a pressure wire, is a necessity, performed only in interventional CCLs, and 36% of UK CCLs are non-interventional.^3^ Second, the skill set, time and equipment must be available to do it. Third, there is a significant upfront cost for each pressure wire. Finally, there are reasons summarised as ‘professional judgement’, where physicians wrongly perceive that they can make the right decisions without the use of physiology.^5^ The solution may lie in the availability of computational physiology by default, alongside the anatomical imaging.

## What is angiographically derived fractional flow reserve (CAG-FFR)?

**Figure 2:**
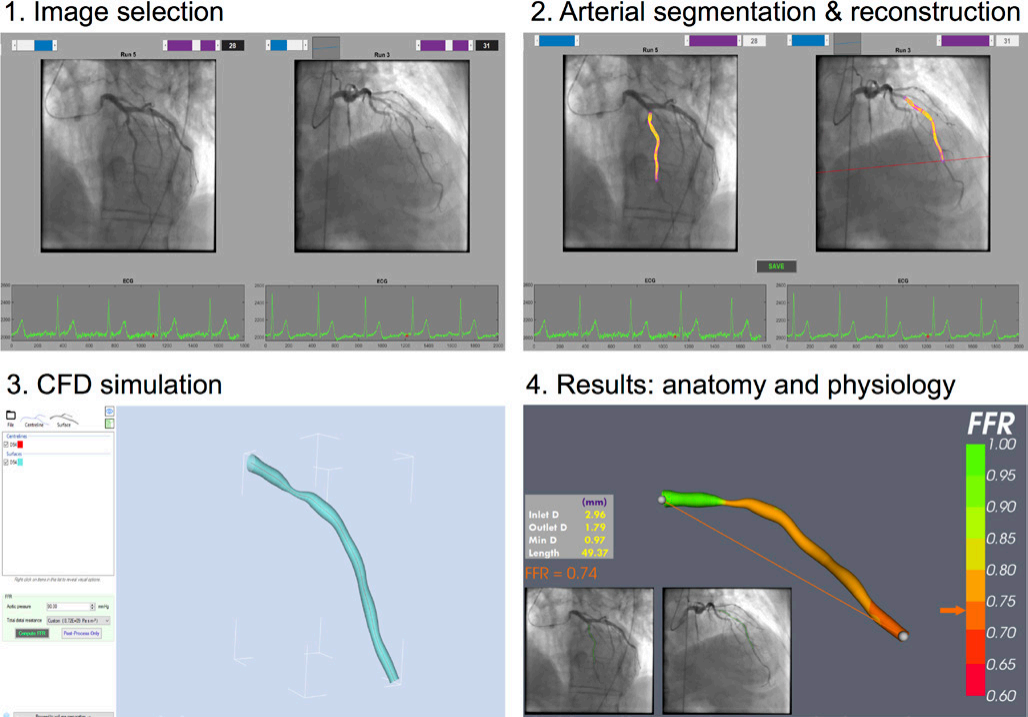
Principal steps in a CAG-FFR workflow. Step 1: optimal views of the lesion are selected with minimal overlap and foreshortening, good opacification, during end diastole, greater than 30° apart; step 2: luminal edge detection and segmentation with 3D reconstruction; step 3: personalised boundary conditions are applied for CFD simulation; step 4: the simulation results are viewed in an interactive graphical user interface providing coregistration of physiology at every point along the modelled anatomy. CAG-FFR, angiographically derived fractional flow reserve; CFD, computational fluid dynamic; FFR, fractional flow reserve.

**Figure 3:**
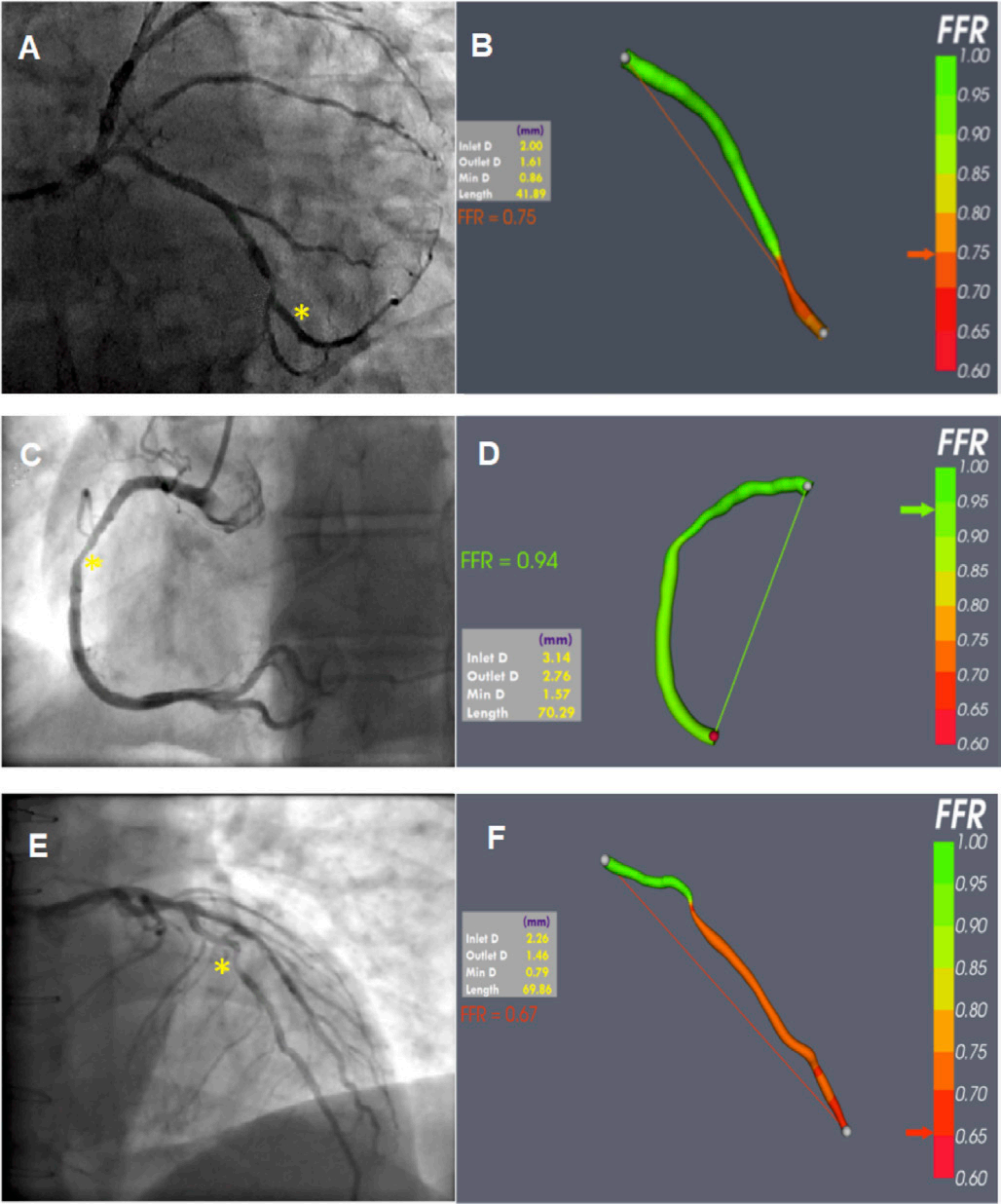
Examples of coronary angiography (left) with corresponding ‘virtual’ FFR results (right). Standard LAO-caudal projection of a distal left circumflex stenosis (*) (A) and VIRTUheart output (B) demonstrating a physiologically significant angiography-derived FFR of 0.75. LAO projection of a mid right coronary stenosis (*) (C) and VIRTUheart output (D) demonstrating a physiologically non-significant angiography-derived FFR of 0.94. Aortic pressure projection of a mid left anterior descending artery stenosis (*) (E) and VIRTUheart output demonstrating an angiography-derived FFR of 0.67 (F), indicating an ischaemia causing lesion. FFR, fractional flow reserve.

CAG-FFR is calculated from medical images of the coronary artery using the physical laws governing fluid flow. The vessel geometry is constructed from either standard CTCA^21–23^ or invasive CAG.^24^ The flow through the reconstructed artery is determined not only by the stenosis geometry but also by boundary conditions that represent the physiological conditions at the inlet, outlets and vessel wall. These can be prescribed to simulate hyperaemic or resting flow conditions. The selection of boundary conditions is an accuracy-defining step.^24^ The CTCA system (HeartFlow Inc, Redwood City, California, USA) is already impacting clinical practice and can be used to improve the role of CTCA as a gatekeeper for CAG, especially when CTCA shows CAD with uncertain functional significance.^1^ However, its availability only as a core laboratory service (HeartFlow, Redwood City, California, USA), lower specificity compared with invasive physiological assessment and image limitations (see further) represent its main limitations.^25 26^ A variety of systems that compute angiography-derived FFR from CAG using different methodologies are now available. Figure 2 outlines the basic workflow of CAG-FFR calculation. The computational time has been reduced to minutes or even seconds, making these systems viable in the CCL. The first of these was the VIRTUheart software developed by the University of Sheffield, employing computational fluid dynamic (CFD) modelling to calculate FFR.^24^ Commercially available systems now include quantitative flow ratio (QFR) (Medis, Leiden, Netherlands, and Pulse Medical Imaging Technology, Shanghai, China), FFRangio (CathWorks, Kfar-Sba, Israel) and CAAS QCA 3D (Pie Medical, Maastricht, Netherlands), which use 3D QCA with mathematical modelling to compute coronary lesion significance. Examples of CAG-FFR results are shown in figure 3.

### Can we rely on CAG-FFR?

To assess CAG-FFR, we must first consider the accuracy of measured FFR because FFR is a surrogate for flow reserve, and CAG-derived FFR is therefore a ‘surrogate of a surrogate’. Table 1 summaries the diagnostic parameters of CAG-FFR from major trials.^24 27–38^ The first difficulty is that FFR is the best tool we have and there is no better test with which to compare it. Second, poor technique can adversely affect its accuracy. It is important that the catheter is not ‘plugged’ in the artery; the wire position is appropriate and stable; and the value recorded is during plateau (stable) hyperaemia. Third, there is variability between repeat measurements, even in the best hands, driven by biological variation. As with other tests of ischaemia, CAG-FFR is assessed against measured FFR, with the limitations outlined earlier. CAG-FFR is not subject to any of the technical limitations of directly measured FFR, but its accuracy is poorest around the threshold of treatment (0.80). The accuracy of CAG-FFR is, however, related to the accuracy of the reconstruction (influenced by the quality of CAG) and to assumptions made in the mathematics applied in the computation of FFR. In the FAVOR (Diagnostic performance of in-procedure-angiography-derived quantitative flow reserve compared to pressure-derived fractional flow reserve) II study, the accuracy of QFR (vs measured FFR) was 71.3% between FFR values of 0.75 and 0.84.^28^ For comparison, the accuracy of CTFFR in the zone 0.70–0.80 is only 46.1%.^39^ The overall limits of agreement (akin to a 95% CI) for CAG-FFR and CTFFR are similar at ±0.14 and ±0.15, respectively. In the context of the clinical range of FFR (0.50–1.00), a CI of ±0.14 is relatively poor,^40^ especially for lesions close to the threshold. Such cases may require a further test of ischaemia, and ideally a directly measured FFR, with the possibility of follow-on PCI. Finally, its performance and accuracy in the hands of operators who are not CFD-modelling experts, beyond special interest research centres, are yet to be established.

**Table 1:** Summary of the major trials reporting the diagnostic performance of angiographically derived FFR

	Software	Accuracy (%)	Sensitivity (%)	Specificity (%)	PPV (%)	NPV (%)	AUC	Patients (n)	Correlation with FFR	BA limits of agreement
Morris *et al* 24	vFFR	97	86	100	100	97	*	19	0.84	FFR±0.16
Tröbs *et al* 30	FFRangio	90	79	94	85	92	0.93	73	0.85	FFR±0.13
Tu *et al* 27	QFR	88	78	93	82	91	0.93	68	0.81	FFR±0.13
Papafaklis *et al* 29	vFAI	88	90	86	80	94	0.92	120	0.78	*
Pellicano *et al* 31	FFRangio	93	88	95	22†	0.12†	0.97	184	0.90	FFR±0.10
Kornowski *et al* 32	FFRangio	94	88	98	*	*	*	88	0.90	FFR±0.10
Xu *et al* 33	QFR	92	95	92	86	97	0.96	308	0.86	FFR±0.13
Yazaki *et al* 34	QFR	89	89	88	74	95	0.93	142	0.80	FFR±0.10
Westra *et al* 28	QFR	83	77	86	75	87	0.86	172	0.70	FFR±0.12
Fearon *et al* 35	FFRangio	92	94	91	89	95	0.80	301	0.80	FFR±0.13
Omori *et al* 36	FFRangio	92	92	92	*	*	0.92	50	0.83	FFR±0.14
Stähli *et al* 37	QFR	93	75	98	89	94	0.86	436	0.82	FFR±0.07
Li *et al* 38	caFFR	96	90	99	97	95	0.98	328	0.89	FFR±0.10

*Not reported.

†Likelihood ratio reported.

AUC, area under the receiver operating curve; BA, Bland-Altman; caFFR, FlashAngio Rainmed, China; FFR, fractional flow reserve; FFRangio, CathWorks, Israel; NPV, negative predictive value; PPV, positive predicted value; QFR, quantitative flow ratio; vFAI, CAAS 3D-QCA, Pie Medical Imaging, Netherlands; vFFR, VIRTUheart, University of Sheffield, UK.

**Table 2:** Comparison of CTCA with invasive CAG

**Factor**	**CTCA**	**Invasive CAG**
Invasiveness	Non-invasive	Invasive
Cost (£)	305*	2000*
Radiation dose (mSv)	2–5	2–12
Contrast dose (mL)	50–120	13–90
Spatial resolution (mm)	0.50	0.16
Temporal resolution (ms)	83–153	1–10
Sensitivity for obstructive CAD	High	Gold-standard investigation
Specificity for obstructive CAD	Low to moderate	Gold-standard investigation
Patient limiting factors	Calcification Tachycardia/irregular heart rhythm Low eGFR	Severe frailty Low eGFR
Other limiting factors	Intolerance of rate-limiting medication Motion artefacts	Intolerance of hyperaemia-inducing medication
Physiological adjuncts	FFRCT	Invasive FFR/iFR/CFR CAG-FFR
Complication rate	Contrast-induced anaphylaxis <1% Contrast-induced nephropathy 3% Side effects related to rate-limiting medications uncommon	Arterial access site complications (radial) 0.2% Major adverse events (MI 0.05%, CVA 0.07%, death 0.08%) Contrast-induced anaphylaxis <1% Contrast-induced nephropathy 3%

*Average cost of a standard outpatient NHS study.

CAD, coronary artery disease; CAG, coronary angiography; CAG-FFR, angiographically derived fractional flow reserve; CTCA, CT coronary angiography; CVA, cerebrovascular accident; eGFR, estimated glomerular filtration rate; FFR, fractional flow reserve; FFRCT, computed tomography derived fractional flow reserve; iFR, instantaneous wave-free ratio; MI, myocardial infarction.

### What are the advantages of CAG-FFR?

CAG-FFR is an ideal all-in-one test for patients being assessed for revascularisation, particularly for those triaged directly for invasive CAG. CAG-FFR could provide enhanced and rapid decision-making while the patient is on the table. Its great advantage is that it can provide a preliminary physiological assessment in any CAG, including in non-tertiary centres, without the need of a wire, an interventionist, extra equipment or expense. This represents a substantially increased potential compared with the present situation. In the UK, for example, of the annual 250 000 CAGs, only about 13 000 include pressure wire assessment, all of which are in interventional CCLs, and of the 100 000 PCIs performed, only 10 000 involve pressure wire assessment.^3^ Therefore, of all patients assessed and treated, invasive physiology is deployed in only 6%–7%. The availability of CAG-FFR is likely to considerably increase the availability of coronary physiology, wherever a CAG be performed, with a reduction in subsequent non-invasive tests of ischaemia, delays and further visits to the CCL. If performed in an interventional CCL, it can justify proceeding to PCI immediately but, importantly, deferring it in others. The software licences for CAG-FFR means the per-patient price will likely be low and, being software-based, can be integrated into existing CCLs relatively simply. CAG-FFR may also enable advanced treatment planning by simulating the physiological effects of virtual stent deployment. This, in turn, could help operators to achieve optimal physiological benefit while minimising the length of the stent deployed.^41^ Ultimately, the CFD methods behind CAG-FFR may also enable quantification of absolute (volumetric) blood flow and other parameters, such as microvascular resistance, providing a more comprehensive coronary physiological assessment.^42^ In the future, intravascular ultrasound and optical coherence tomography, coregistered with CAG, may augment anatomical reconstruction, and this may improve CAG-FFR accuracy.

### How does CAG-FFR compare with other tests of ischaemia?

When invasively measured FFR was introduced, it was validated against the accepted tests of the day: exercise testing, thallium single-photon emission CT (SPECT) and stress echocardiography.^8^ Since then, stress perfusion cardiac MRI (CMR) has become established as superior to SPECT.^43^ In the CE-MARC trial, FFR was used as the reference standard, against which perfusion MRI was assessed.^43^ So, when CAG-FFR is assessed against SPECT, there is a degree of discordance, but, compared with perfusion CMR, its accuracy is 92%.^44^ It is important to appreciate the differences between these tests. The main one is that CMR is an excellent test of the overall and regional burden of ischaemia, whereas FFR addresses the contribution of a specific lesion in a particular artery. The other is that FFR, while being accurate as regards the contribution of the lesion to blood flow limitation, provides no information about the state of the microvasculature.^42^ Indeed, disease in this compartment may explain some of the ‘false positive’ results of non-invasive tests like pMRI when compared with CAG and FFR. Although primarily an anatomical test, like CAG, CTCA is the basis of CTFFR. Compared with invasive FFR, accuracy is approximately 84%.^21 22^ The limitations of CTFFR are those of CT itself, namely, image resolution (still considerably lower than CAG), calcification, atrial fibrillation, tachycardia and motion artefact contributing to its wide zone of uncertainty around the clinical decision point of 0.80, which is exactly where precision is required.^39 45^ As with FFR, neither CAG-FFR nor CTFFR provide information about absolute blood flow or microvascular physiology.

### What are the limitations of CAG-FFR?

Like CTFFR, CAG-FFR is particularly dependent on the quality of the angiographic images. This is because CAG is essentially a series of two-dimensional (2D) images that need to be converted into a three-dimensional (3D) computational model. Even a straight tube with a simple stenosis needs two 2D images, at least 30 degrees apart, to derive a reasonably accurate 3D model. Thus, lesions located at bifurcations, with an overlapping vessel, at the arterial ostium or in the left main artery, pose particular challenges. As with a simple diagnostic CAG, poor catheter engagement, inadequate artery opacification with contrast, excessive ‘panning’, movement (patient, respiratory or cardiac), magnification or ‘coning’ that obscures or cuts off parts of the vessel are problematic. Therefore, as many as 80% of CAGs are unsuitable for analysis, but with some simple improvements in angiographic technique (box 1), this figure can be substantially reduced.^46^ Centres that have adopted CAG-FFR often report improvement in the quality of angiography when working with an acquisition protocol suited for CAG-FFR analysis. A level of skill in image processing is also required, with knowledge of coronary anatomy and training in using the software, particularly at the segmentation step.^47^ In most centres, this is likely to be the domain of the radiographer. The main scientific limitation and challenge in these models is that of variability in the resistance of the coronary microvascular bed. Not only is this the dominant influence of coronary blood flow and FFR, but also it is the single largest contributor to error in CAG-FFR.^48^ Because microvascular resistance is unknown, models rely on assumptions which do not apply in all patients, such as those with prior MI, diabetes or LVH.^49^

Box 1 **Standardised angiography protocol to maximise the applicability of angiographically derived fractional flow reserve**

**General measures**

- Administer Glyceryl trinitrate prior to acquisition to minimise spasm.
- Centre the image before acquiring.
- Minimal magnification (mag) (+1 mag only if small patient).
- Minimise table movement ('panning').
- Increase X-ray dose if the patient is obese.
- Good catheter engagement.
- Good opacification of the vessel.
- Acquisition over at least four cardiac cycles.
- Minimal adjustment of table height between runs.
- Ensure ECG signal is captured (for ECG gating in some systems).

**Suggested RCA views**

- LAO cranial.
- PA cranial.
- RAO cranial.

**Suggested LCA views**

- PA caudal
- RAO caudal.
- PA cranial
- LAO caudal (40°/40°).

Ensure good separation between projections (at least 30°), good visualisation of the lesion with minimal overlap or foreshortening of vessels.

### Where could CAG-FFR fit into future practice?

**Figure 4:**
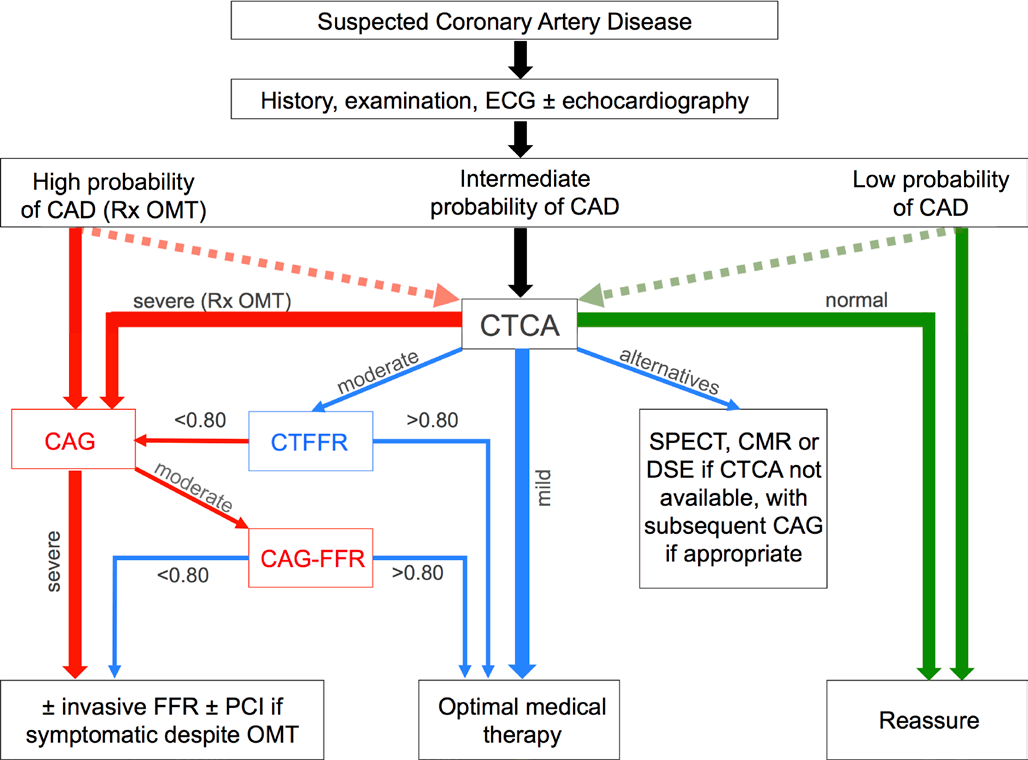
Proposed algorithm for the diagnostic pathway of suspected CAD integrating CTFFR and CAG-FFR. CAD, coronary artery disease; CAG, coronary angiography; CMR, cardiac MRI; CTCA, CT coronary angiography; CTFFR, CT fractional flow reserve; FFR, FFR, fractional flow reserve; OMT, optimal medical therapy; PCI, percutaneous coronary intervention; SPECT, single-photon emission. DSE, dobutamine stress echocardiography.

In the same time frame as physiological assessment became acknowledged to be superior to solely anatomical assessment of coronary heart disease, UK guidelines changed to advocate CTCA, a purely anatomical test. Functional tests of ischaemia are recommended in cases in which there is uncertainty about the findings of CTCA.^1^ These same tests are also first-line investigations in symptomatic patients with confirmed CAD. CAG is currently only recommended as a third-line investigation. Pretest stratification according to the likelihood of significant CAD often overestimates risk and has fallen out of current national guidance.^3^ European guidance supports the use of CAG for patients who have a high pretest probability of CAD with significant risk factors and refractory angina or typical angina at low workloads,^8^ a pathway which retains some popularity for many cardiologists. Given the characteristics of CTCA and CAG (table 2), current uncertainties and the variability of locally available investigations, we propose a modified algorithm for the diagnosis of obstructive CAD incorporating virtual coronary physiology (figure 4). In this framework, the role of CAG is strengthened. Instead of some patients at medium and higher risk requiring both a non-invasive test and CAG, they could have a stand-alone CAG-FFR providing a detailed and appropriate plan for revascularisation in a time-efficient manner. Several clinical trials are currently ongoing to evaluate the impact of CAG-FFR on clinical outcomes.^50^

## Conclusion

The addition of computational modelling of blood flow to a standard CAG can provide a detailed and specific ‘all-in-one’ combined anatomical and physiological assessment of CAD at a low cost. It could help guide decisions about revascularisation, streamline management, be a useful gatekeeper to the interventional laboratory, and triage patients and lesions for direct, invasive measurements of FFR and similar indices. The lack of requirement for a pressure wire makes this technology feasible in the purely diagnostic cardiac catheterisation laboratory, providing the benefits of physiological guidance to a far greater number of patients with CAD than at present receive it (Box 2). Angiography-derived physiology may represent a renaissance for invasive CAG.

Box 2 **Summary of key points**

**What do we already know?**

- Despite non-invasive investigations, invasive coronary angiography (CAG) remains the final common investigation for all patients under assessment for revascularisation.
- Visual assessment of stenosis severity is subjective and its relationship to ischaemia is unreliable.
- FFR is the gold standard for invasive ischaemia testing, but is under-used.

**Key learning points:**

- Some systems of ‘virtual’ FFR, based on invasive CAG, are now approved for clinical use and could provide an ‘all-in-one’ test for coronary artery disease.
- The accuracy of CAG-FFR depends on good quality image acquisition and optimal technique.
- CAG-FFR can be integrated into existing cardiac catheter laboratories and may expedite and simplify patient assessment.
